# Development of a Chitosan-Based Biofoam: Application to the Processing of a Porous Ceramic Material

**DOI:** 10.3390/ijms12021175

**Published:** 2011-02-16

**Authors:** Jean-Denis Mathias, Nicolas Tessier-Doyen, Philippe Michaud

**Affiliations:** 1 Laboratoire d’Ingénierie pour les Systèmes Complexes, CEMAGREF, Campus des Cézeaux 24 avenue des Landais—BP 50085, 63172 Aubière Cedex, France; 2 Laboratoire Groupe d’Etude des Matériaux Hétérogènes (GEMH—ENSCI EA 3178) Centre Européen de la Céramique—12 rue Atlantis, 87068 Limoges Cedex, France; E-Mail: nicolas.tessier-doyen@unilim.fr; 3 Clermont Université, Laboratoire de Génie Chimique et Biochimique, Université Blaise Pascal, Polytech’ Clermont Ferrand, 24 avenue des Landais BP 206, 63174 Aubière cedex, France; E-Mail: Philippe.michaud@univ-bpclermont.fr

**Keywords:** chitosan, biofoam, ceramic slurry

## Abstract

Developing biofoams constitutes a challenging issue for several applications. The present study focuses on the development of a chitosan-based biofoam. Solutions of chitosan in acetic acid were dried under vacuum to generate foams with high-order structures. Chitosan concentration influenced significantly the morphology of developed porosity and the organization of pores in the material. Physico-chemical characterizations were performed to investigate the effects of chitosan concentration on density and thermal conductivity of foams. Even if chitosan-based biofoams exhibit interesting insulating properties (typically around 0.06 W·m^−1^·K^−1^), it has been shown that their durabilities are limited when submitted to a wet media. So, a way of application consists to elaborate a ceramic material with open porosity from a slurry prepared with an organic solvent infiltrating the porous network of the foam.

## 1. Introduction

Plastic foams have been extensively employed in a variety of applications, such as thermal insulation, weight reduction, packaging or open-cell cellular ceramic materials manufacturing. These foams are made of different plastic materials such as polystyrene, polyurethane or polyvinyl chloride. In 1998, 2.5 billion kg of plastic foams were produced [1]. Industrial foams are mainly based on depleting petroleum resources. Despite a little part of these foams being recycled, their intensive usage runs counter to environmental- and climate-friendly development, as petroleum is not a renewable resource. Developing biosourced-foam has been addressed in the literature since the 1990s. Some studies have consisted of using starch in order to replace industrial foams. Indeed, starch has the advantage of being cheap and abundant, facilitating its industrial development. For example, Wang *et al.* [2] studied the use of wheat and corn starches in order to develop a new generation of biofoam. Lin *et al.* [3] employed amylose cornstarch for packaging applications. Different studies have associated starch with plastic components so as to reduce the environmental impact. For example, Bhatnagar *et al.* [4–6] associated the amylose cornstarch with polystyrene and polymethyl methacrylate. Lay *et al.* [7] coupled the use of starch with polystyrene and Fang and Hanna studied copolyester-starch based foam [8]. Behling [9] developed a soy-based rigid polyurethane foam for thermal insulation applications. This soy-based foam was made with soybean flour with water used as a blowing agent. Guo *et al.* [10] also investigated the use of soy to product biofoam. Guo *et al.* developed rigid polyurethane foams based on soybean oil [10]. Foams have been prepared from polyols derived from soybean oil and the effect of formulation linked to foam properties was studied by altering the types and amounts of catalyst, surfactant, water, crosslinker, blowing agent, and isocyanate, respectively. Other research based on vegetable oils has been done in order to replace petrochemicals. The idea was to convert vegetable oils into polyols for polyurethane foams, such as palm oil [11] or castor oil [12]. The main drawbacks limiting the development of those biofoams were their rapid biodegradability and the consumption of raw materials initially produced for food.

At the same time, the use of chitosan has been wide-spread in several applications. This polysaccharide is obtained by alkaline deacetylation of chitin, which is the main component of the exoskeleton of crustaceans [13]. It is wide-spread, abundant and its production is cheap and ecologically interesting [14]. Moreover, this polymer obtained from by-products of crustacean exploitation has interesting antimicrobial properties. Recent research devoted to chitosan has been done in order to form starch citrate-chitosan foams [15].

The aim of this study was to determine the physical characteristics of chitosan-based foam. It especially focused on the influence of chitosan concentration on foam properties in terms of microstructure, porosity rate and thermal conductivity. The main results dealt with the fact that chitosan concentration induced significant variations in the microstructure and, therefore, modifications of the thermophysical macroscopic properties of the biofoam. Due to the sensibility of the biofoam to water, the explored way of application consisted of using the replica technique with the developed foam acting as a template for the impregnation of a ceramic slurry. Indeed, foams used for this type of impregnation are based on non-renewable resources and the proposed biofoam is relevant for this type of application. The first section describes the manufacturing technique of the foam and the testing methods investigated to characterize the developed biofoam. The second section is devoted to the results of physical properties analyzed by employing testing methods with respect to the chitosan concentration. The third section focuses on the use of the biofoam as a template of infiltration for a ceramic slurry.

## 2. Experimental Methods

### 2.1. Material

Chitosan is a polymer of β-(1,4)-linked 2-acetamido-2-deoxy-d-glucopyranose and 2-amino-2-deoxy-D-glucopyranose. This polycationic biopolymer is generally obtained by alkaline deacetylation of chitin, which is the main component of the exoskeleton of crustaceans [13]. Chitosan is the sole cationic polysaccharide due to its positive charges (NH_3_^+^) at acidic pH (pH < 7). These charges increase retention at the site of application [16]. The main parameters influencing the attractive characteristics of chitosan are its molecular weight (Mw) and degree of deacetylation (DD). 75% deacetylated commercial chitosan powder (Sigma Aldrich C3646) was used for biofoam formulations. The degree of acetylation may be determined by dye binding [17] or titration methods [18] according to the supplier. 1–4% chitosan solutions were made in 1% acetic acid (Ac). The dissolved chitosan solution was left for 24 h at 50 °C prior to being applied to remove bubbles. Different chitosan concentrations were chosen to investigate different experimentations. The objective was to investigate the influence of the chitosan on the biofoam properties. Samples (50 mL) were incubated 12 h at −80 °C in a cylinder sample bottle in polystyrene (50 × 65 mm^2^) before freeze drying.

### 2.2. Preparation of the Foam Samples

In order to manufacture biofoams, the chitosan solution was freeze dried for 55 hours. Obtained samples were cylinders with an approximate size of 47.5 mm diameter and nearly 38.5 mm height. Elaborated samples exhibited shrinkage of about 27% in the direction of the diameter and 23% in the direction of the height.

### 2.3. Testing Methods

Characterizing the physical properties of the chitosan-based bioafoam is of prime importance in order to use it for suitable applications. Several tests were carried out, so as to link the influence of chitosan concentration to macroscopic properties.

The main aim of these experiments was to study the structure with respect to chitosan concentration. For this purpose, optical observations were investigated using an optical microscope (Eclipse E200, Nikon) instrumented with low values of zoom (×4 and ×10) so as to have a global description of the biofoam structure.

Then, a scanning electron microscope (SEM) was employed to assess the microstructure of the foam in function of the chitosan concentration. The SEM enabled us to obtain images of sample surfaces by scanning it with a high energy beam of electrons in a raster scan pattern. Furthermore, the zoom reached an amplification of 400 which allowed a more accurate observation at a lower scale of the microstructure. For this purpose, specimens were cut in smaller samples. A fine deposition of gold-palladium on the surface of samples gave sufficient electrical conductivities to allow observations.

The porosity of the foam was evaluated using two complementary methods. The first one consisted of measuring mass and typical dimensions of samples considered to be perfect cylinders to obtain the apparent density ρapp (solid phase + porosity). From these results, it was possible to evaluate the overall porosity rate (π) with the knowledge of the density of the foam solid structure *(*ρ_*solid*_) measured by helium pycnometer (AccuPyc 1330, Micromeritics):

(1) $$\pi =1-\frac{\rho_{\text{app}}}{\rho_{\text{solid}}}$$

Finally, thermal conductivities of the different biofoams were measured in two directions (in directions parallel and perpendicular to the axis of the cylindrical samples) with steady state heat flow equipment operating at room temperature (CAPTEC). In order to carry out thermal measurements, 3 samples (30 × 30 mm^2^) with 3 different thicknesses (from 2 to 5 mm) were cut from the previous cylindrical block sample. A difference of temperature (ΔT) equal to about 5 °C was imposed across the sample. The resulting average heat flow (φ_m_) through the investigated sample was determined using thermoelectric heat flux sensors to assess the incoming and outgoing heat flows. The apparent thermal resistance (*R_app_*) of the sample was then calculated as [19]:

(2) $$R_{\text{app}}=\frac{\Delta T}{\varphi_{m}}=\frac{e}{\lambda }+R_{\text{contacts}}$$

Where *e* is the typical thickness, and *R_contacts_*, the overall resistance of the contacts of the studied sample to the copper plates of the sample holder. The advantage of investigating three different thicknesses of samples was that it was possible to avoid the contribution (*R_contacts_*) in apparent thermal resistance (*R_app_*). Indeed, least squares linear regression can be used for experimental data plotted in the form of thickness (*e*) *versus R_app_* to calculate the value of the thermal conductivity (λ) from the slope.

## 3. Physical Characterization Results

### 3.1. Effect of Chitosan Concentration on the Microstructure

In a first approximation, it can be observed that the porous specimen is not perfectly homogeneous in its whole volume. In fact, the bottom and the edges of the sample seem to exhibit less porosity rate than the core and the top. This may be due to the surface tension of the polymer on the edges of the container during the preparation.

**Figure 1.**
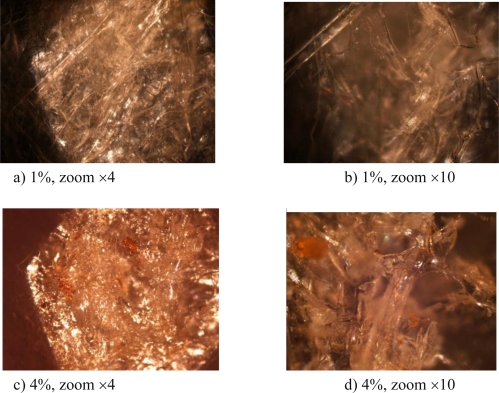
Optical microscope observations of the biofoam microstructure. The 1% biofoam (**(a)** and **(b)**) exhibits more filament particles than the 4% biofoam (**(c)** and **(d)**), but is less compact.

The chitosan concentration strongly influenced the microstructure and it especially changed the internal organization of the foam’s structure. Figure 1 shows the microstructure of the foam for two concentrations: 1% and 4%.

**Figure 2.**
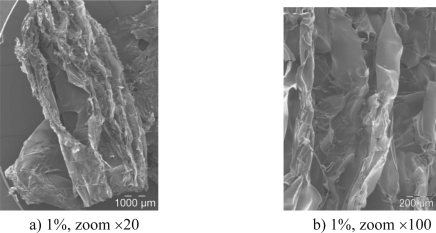

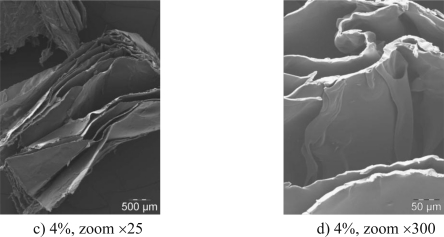
Scanning electron microscope (SEM) observations of the biofoam. **(a)** and **(b)**, The 1% biofoam exhibits filaments; **(c)** and **(d)**, the 4% biofoam presents a leaf-based structure.

The optical microscopy enabled assessment of the microstructure of the biofoam which was composed of entangled filaments or leaves. The 1% biofoam appeared to be less compact than the 4% biofoam which may have been due to a dominating filament-based microstructure. In order to investigate this point in more detail, the SEM results are presented in Figure 2.

The SEM images confirmed the conclusions of the OM images because the structures of the two biofoams were significantly different. Indeed, the 1% chitosan biofoam was composed of entangled filaments with no significant ordered structure, whereas the 4% chitosan biofoam exhibited organized leaves. Furthermore, it seemed that the leaves were oriented in a preferred direction because the planes of the leaves seemed to be parallel. So chitosan concentration introduced in rather small quantities had a great impact on the organization of the biofoam microstructure during the freeze drying. The filamented structure seemed to favor the amount of voids in comparison to the leaf structure. It is interesting to note that in the study of Salam *et al.* [15], the observed structure of starch citrate-chitosan foam was close to the present 1% chitosan foam structure. However, this point has seldom been addressed in [15].

To evaluate the influence of chitosan concentration in relation to the microstructure of foams on their thermophysical properties, density and thermal conductivity, measurements were investigated and are presented in the following section.

### 3.2. Effect of Chitosan Concentration on the Density

The solid and apparent densities of the biofoam were evaluated for chitosan concentration of: 1%, 3% and 4%. Four specimens were tested per concentration after drying at 60 °C for 24 h. The mean value of the foam solid density determined with a helium pycnometer was equal to 1.15 ± 0.04 g·cm^3^. Apparent density measurements and calculated porosity rates are reported in Table 1.

The densities of samples were very low for such material and the lower the chitosan concentration, the lower the density. The calculated porosity rate was more important for the 1% concentration (96%) than for the other two chitosan concentrations which remained almost the same. These results are in agreement with the observations performed in the previous section concerning the microstructure of the biofoam.

### 3.3. Effect of Chitosan Concentration on Thermal Conductivity

**Figure 3.**
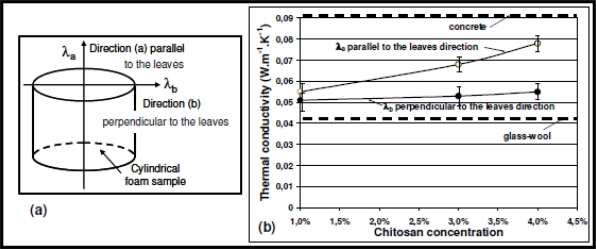
**(a)** Representation of directions (a) and (b) of the thermal conductivity; **(b)** Results of thermal conductivity measured in the two directions with respect to chitosan concentration.

Thermal conductivities of the biofoams were measured in two directions [a] and [b] respectively parallel (λ_a_) and perpendicular (λ_b_) to the orientation of leaves. Three concentrations of chitosan were studied: 1%, 3% and 4% and four specimens per concentration were tested. Results are reported in Figure 3.

It can be observed that the thermal conductivity increased with chitosan concentration. Moreover, in the parallel direction to the leaf orientation, the values were slightly higher than in the perpendicular direction. The mean scattering of values was determined to be less than 0.004 W·m^−1^·K^−1^ which represents about 10% of the mean values. For the lowest chitosan concentration, the thermal conductivity values λ_a_ and λ_b_ were quite the same whatever the direction of measurement, whereas the difference was significant for the highest concentrations. These results are in agreement with SEM observations. The preferential orientation of leaves (3% and 4% chitosan concentration) in the parallel direction to the axis of the cylindrical samples contributed to increase the overall thermal conductivity of samples whereas for disorganized structure with non-ordered filaments (1% chitosan concentration), there was no effect of microstructure. The variation of porosity rate had no significant influence on λ_b_ values which remained quite stable. The trend in that direction was explained by the neutralization of two effects: (i) the evolution of open pores distribution from not organized individual cells to a quite oriented porous network, which contributed to a decrease in the overall λ_b_ value; (ii) the rate decrease of porosity which contributes to increase the overall λ_b_ value.

These foams exhibited low density and thermal conductivity values (around 0.06 W·m^−1^·K^−1^) rather close to those of insulating glass-wool materials (around 0.045 W·m^−1^·K^−1^). So, they can be considered as potential candidates for packaging or insulating applications.

### 3.4. Influence of a Wet Media

**Figure 4.**
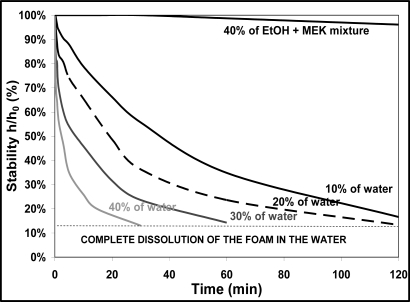
Evolution of the sample’s height submitted to a wet media in function of time.

In order to test the durability of foams as a function of time, cylindrical samples of a 1% chitosan composition were submitted to a wet media. The test consisted of putting the sample in a container with a fixed quantity of water to evaluate the water stability. As the water was completely absorbed by the open pores, the sample changed due to the water absorption by the solid skeleton. Different volume proportions of water of the total volume (sample + water) were tested to follow the stability of foams as a function of time (up to 2 h). In Figure 4, the chosen characteristic parameter of stability was obtained by measuring the height (h) of the sample at a time t out of to the initial height (h_0_).

In contact with water, the foam quickly became a gel because of all the water it absorbed. The velocity of gelation was even more important, as the initial quantity of added water was high; so the foam seemed to be instable in water. In contact with a binary azeotropic solvent (mixture composed of 40% ethyl alcohol (EtOH) and 60% methyl ethyl ketone (MEK)), the foam kept a rather good integrity for two hours.

These results suggest that even if the density and thermal conductivity are competitive in regard to other common materials, this type of chitosan-based foam is not adapted for insulating and packaging applications because of its weak resistance to humidity. The current biofoam can be associated and formulated with other materials in order to enhance its stability in water. However, the present paper focuses on chitosan-based biofoam, so it was decided to use the biofoam for a relevant application, that is, the impregnation of ceramic slurry. Such an application is described in the following section.

## 4. Application: Elaboration of a Porous Ceramic Material

### 4.1. Principles of Replica Technique

Another application consists of using this foam as a template for impregnation of a ceramic slurry [20,21]. The final goal was to elaborate a porous ceramic material with open porosity after the burn out of the fugitive developed biofoam. Indeed, the burned out conventional foams used for this application (e.g., polyurethane) gave off some non-environmentally friendly byproducts. Therefore, it seems more relevant to use the current biofoam for this application.

**Figure 5.**
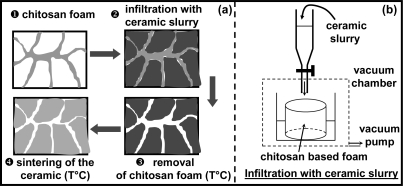
**(a)** Schematic representation of fabrication process of the porous ceramic from the chitosan foam; **(b)** Details of the slurry infiltration apparatus.

The different steps of the processing are described in Figure 5a. The open pores of the foam were filled by a ceramic slurry introduced in a vacuum chamber (Step 2).

In step 3, the filled foam was dried and slowly centrifuged to eliminate the excess impregnated slurry. Then, the polymer part was removed by thermal treatment. The final step concerned the sintering of the ceramic material exhibiting open porosity rate whose shape was close to that of the solid skeleton of the initially infiltrated chitosan foam.

### 4.2. Preparation of the Ceramic Slurry

A stabilized ceramic slurry [22] was obtained starting from fine α-alumina powder (Alcoa A-16SG, φ_50_ ≈ 0.5 μm). The main steps of the experimental procedure are described in detail elsewhere [23,24]. The organic solvent (mixture composed of 40% ethyl alcohol (EtOH) and 60% methyl ethyl ketone (MEK)) was mixed with the powder and a phosphate ester (dispersant) over a 24 hours period. Binder (PVB) and plasticizer (PEG) were then introduced and the overall mixture was milled for 12 hours. The proportion of ceramic powder of the total weight of mixture (powder + organic additives) was about 55%. This proportion (indicating that the suspension was not too concentrated) was chosen in order to facilitate entrance of the slurry in the porosity of the template.

### 4.3. Infiltration of the Foam

Then the slurry was introduced in the vacuum chamber in order to fill the open porosity of a 4% chitosan foam sample at a stabilized pressure of 32 mBars. It was observed that the slurry exhibited quite a good wetting behavior of the chitosan foam template. At the end of the infiltration, the impregnated foam was removed from the chamber. It was then centrifuged at 1000 rpm during 1 min and dried at room temperature for 1 h. This procedure was repeated three times to improve the thickness of the deposit all around the surface of the leaves in the foam template.

### 4.4. Pyrolytic Decomposition of the Chitosan Foam

**Figure 6.**
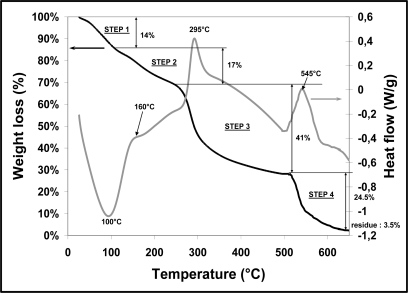
Thermal behavior (mass loss and heat flow) of the foam during a thermal cycle.

After drying at room temperature for 48 h, the infiltrated foam was submitted to a thermal cycle (0.2 °C/min up to 600 °C) to remove water and all the organic phase (foam + slurry organic additives). To have a better understanding of the critical steps occurring during the pyrolytic decomposition of the chitosan foam, a small quantity (about 20 mg) was analyzed at 10 °C/min up to 650 °C in a helium atmosphere (continuous gas flow maintained at 10 mL/min) with a thermogravimetric apparatus (ta-instruments, SDT Q600). This device enabled us to follow the percentage of mass loss *versus* temperature and the variations of thermal heat flow. The curves are given in Figure 6.

The curve of weight loss coupled with the heat flow curve, exhibited four steps on increasing the temperature in non-oxidized conditions (helium atmosphere): a peak temperature appeared at about 100 °C, corresponding to an endothermic pic (mass loss is about 14%) and was attributed to the evaporation of water adsorbed and/or weakly hydrogen-bonded water. The second weight loss of 17% at about 160 °C was probably due to linked water (hydrogen bonded water) in the structure of the foam. Then the significant mass loss at about 300 °C (41%) was probably due to the depolymerization of the chitosan and decomposition of pyranose rings, whereas the last step at about 550–600 °C occurred due to the thermal decomposition of char chitosan. Zawadzki *et al.* [25] observed the same phenomenon during thermal degradation of chitosan in N_2_ and explained it by a free radical mechanism in which intermediate radicals recombine to generate crosslinked networks more stable to temperature. At 650 °C, a small quantity of carbonic residue corresponding to 3.5% of the initial mass was obtained.

### 4.5. Sintering of the Green Ceramic Body

After 600 °C, the green alumina powder body was sintered at a fast heating rate of 5 °C·min^−1^ up to 1550 °C for two hours. After the cooling stage to room temperature, the ceramic material exhibited an open porosity rate of about 70% and a mechanical resistance of the material sufficiently high to be manipulated.

## 4. Conclusion

A chitosan-based biofoam was developed. It has been shown that the chitosan concentration has an important influence on the microstructure of the biofoam and therefore on its physical properties. Low chitosan concentrations lead to a filament structure with a lot of voids. It leads to a low value of thermal conductivity, close to the thermal conductivity of glass-wool. When the chitosan concentration was increased, the microstructure of the chitosan changed, leading to a higher value of thermal conductivity. However, it has been highlighted that the biofoam was not stable in the presence of water. Some developments are, however, possible in order to enhance this property by formulation with other materials. One application consists in using the chitosan-based biofoam for the impregnation of a ceramic slurry. We successfully prepared a porous ceramic structure in alumina exhibiting 70% of open porosity using a replica technique. This biofoam seemed to be relevant for this type of application and more environmentally friendly than traditional polyurethane foams when pyrolyzed. However, in order to elaborate highly controlled porosity ceramic materials with these chitosan biofoams, the future of this work lies in the control of the homogeneity of samples (pore size, porosity rate and homogeneity in the whole volume).

## Figures and Tables

**Table 1.** Summary of measured thermo-physical properties of foams in function of chitosan concentration.

Chitosan concentration	1%	3%	4%
Apparent density ρ_app_ (g·cm^−3^)	0.043	0.081	0.088
Overall porosity rate π	96%	93%	92%
Thermal conductivity λ_a_ measured in direction [a] (W·m^−1^·K^−1^)	0.055	0.068	0.078
Thermal conductivity λ_b_ measured in direction [b] (W·m^−1^·K^−1^)	0.051	0.052	0.054
